# Supplementary material for: Are carbon emissions trading and green financial instruments synergistic? -Comprehensive quantitative research based on content analysis
Source: PLoS One. 2024 Mar 7;19(3):e0298601. doi: 10.1371/journal.pone.0298601 (PMC10919601; doi:10.1371/journal.pone.0298601)
Supplement: S1 Data — (ZIP) [file pone.0298601.s001.zip › Data/地级市Z_F工作报告.zip!/║╙─╧/╨φ▓2╩╨/└┤╫╘╣1⁄2╓┌║┼í╛┬φ┐╦╩2╛▌═°í┐_2015.docx]

各位代表：

现在，我代表市人民政府，向大会报告工作，请予审议，并请市政协各位委员提出意见。

一、2014年工作回顾

2014年，面对复杂严峻的经济形势，在省委、省政府和市委的正确领导下，市政府团结带领全市人民，认真贯彻党的十八大及十八届三中、四中全会精神，围绕致力打通“六路”、建设“五型许昌”，统筹推进稳增长、调结构、促改革、惠民生各项工作，经济社会发展呈现稳中有进、稳中向好的态势。全年完成生产总值2108亿元，增长9.3%；地方一般公共预算收入125亿元，增长15.4%；规模以上工业增加值1201亿元，增长11.9%；固定资产投资1637亿元，增长19.4%；社会消费品零售总额619亿元，增长12.7%。一年来，我们主要做了以下重点工作：

（一）全力以赴稳增长，经济实现平稳较快发展。面对经济下行压力加大态势，我们多管齐下、动态调控、稳定增长，主要经济指标增速高于全省平均水平。强化有效投资，实施“15616”投资促进计划，151个重点项目完成投资661亿元，带动全社会固定资产投资1600亿元以上，重点项目对投资增长贡献率超过40%，三大水利、三国文化产业园等重大项目进展顺利。稳定增长主体，促进工业平稳运行，完善服务企业长效机制，定期开展政策咨询会和产销对接、产业链对接、银企对接、用工对接等活动，有针对性地解决了一批企业突出问题。扩大开放招商，实施“6612”招商引资行动计划，通过三国文化旅游周等重大招商活动，合同引进市外资金1260亿元。直接融资96.6亿元，3家企业“新三板”挂牌，2家企业上海股权交易中心挂牌。防范金融风险，稳定企业发展，建立了企业发展互助资金、担保体系，及时打出金融“组合拳”，社会融资规模稳步扩大，信贷投放稳步增长，余额存贷比、新增存贷比分别高出全省11个和43个百分点，分居全省第3位和第2位，荣获全省“优秀金融生态省辖市”称号，金融竞争力居全省第3位。

（二）突出重点调结构，发展质量效益稳步提高。我们把调结构、转方式摆上更加重要位置，着力推动绿色低碳协调可持续发展，转型升级步伐加快，绝大多数效益指标保持全省靠前位次。大力发展高成长服务业，41个重点服务业项目超额完成年度计划，商务中心区、特色商业区重点项目完成投资59.5亿元、增长31.3%，服务业增加值增长9%，魏都特色商业区获全省综合考评第3名。着力做优做强工业，加快产业集聚区建设，大力实施“十百千”培育工程，全市规模以上工业利润和主营业务收入分别完成425亿元、4960亿元，分居全省第2位、第3位；5个省级产业集聚区晋级为星级产业集聚区，主营业务收入超500亿元产业集聚区达到3个、超百亿元产业集群达到9个，装备制造业集群主营业务收入突破千亿元；主营业务收入超百亿元企业达到7家；许继进入“中国电气工业竞争力10强”；黄河、众品、森源、金汇4家企业入选中国民营企业500强，占全省总数的1/3。加快发展现代农业，扎实推进高标准粮田“百千万”、农业产业化集群、都市生态农业三大工程建设，新增高标准粮田40万亩，建成高效节水灌溉面积12.9万亩，粮食产量在遭受历史罕见严重旱灾的年景，仍保持280万吨以上。土地流转面积累计达到154万亩，占家庭承包耕地总面积的37%。省重点农业龙头企业发展到51家，居全省第3位。

（三）科学发展重统筹，城乡一体化进程全面加快。我们研究出台了《许昌市贯彻省科学推进新型城镇化三年行动计划实施意见》，突出城乡一体化示范区，着力六大区域综合开发，提升规划水平，加强顶层设计，完善体制机制，统筹推进城乡发展。“1665”行动计划扎实推进，163个市区城建重点项目、64个六大区域开发建设项目，均超额完成年度投资计划，51个棚户区改造取得新进展，学院路北延等13条道路和许昌东站长途汽车站、新兴公交停保场等建成投用，城市河流清洁行动顺利启动，中心城区河湖水系连通项目全面开工建设。市城乡一体化示范区投资促进计划超额完成，示范区主中心和中原电气谷核心区基础设施进一步完善。经过15年的不懈努力，许昌以全国地级市第4名荣膺“全国文明城市”称号，创建成果惠及千家万户。省级生态文明城市先行示范市申报获得成功，数字化城市管理项目荣获中国人居环境范例奖，禹州市被确定为全国首批新型城镇化试点城市。加快改善农村人居环境，1804个村完成村庄环境整治任务，25个村的扶贫开发整村推进项目全面完工，新解决农村39.2万人安全饮水问题，2.8万农村贫困人口实现稳定脱贫。

（四）强化创新增优势，发展后劲不断得到提升。我们突出以企业为主体的科技创新，科技创新奖励资金达1583万元，国家创新型试点城市创建取得阶段性成效，知识产权试点城市通过验收，实施省级以上重大科技专项和高新技术产业化项目8个，新增11家高新技术企业，新建12家省级工程技术研究中心，高新技术企业和省级工程技术研究中心数量分居全省第4位和第3位，专利申请量和授权量均居全省第3位。推进实施质量立市、国家商标战略，新增省名牌产品数量居全省第2位，中国驰名商标、地理标志证明商标总量分居全省第3位和第4位。着力推进信息化与经济社会融合发展，智慧城市等建设取得新进展，信息化基础设施日益完善，应用水平不断提高。加快生态建设，实施蓝天、碧水、乡村清洁工程，清潩河综合治理进展顺利，长葛市双洎河国家湿地公园获批建设，大周“城市矿产基地”成为国家循环经济教育示范基地，节能减排和单位生产总值能耗下降率完成省定任务。

（五）全面深入促改革，发展动力活力显著增强。我们突出抓好重点领域和关键环节改革，通过改革释放市场活力。深入清理规范行政审批事项和行政事业性收费，优化审批流程，精简审批环节，压缩审批时限，实现了市级行政审批项目全省最少、审批时限全省最短。扎实推进企事业单位改革改制、“营改增”试点、商事制度等改革，4家单位完成改制任务，新登记各类市场主体超过2.5万户、新增注册资本超过320亿元。加快开放平台建设，设立许昌海关获得批准，许昌出入境检验检疫局正式挂牌开检。

（六）加大力度惠民生，人民幸福指数持续提高。我们坚持以人为本，加大民生投入，“十项重点民生工程”全面落实，全市涉及民生的财政支出达157.7亿元、增长8.2%，占一般公共预算支出的71.3%。城镇居民人均可支配收入23753元、增长9.4%，农民人均纯收入12140元、增长10.3%，新增城镇就业9.4万人、农村劳动力转移就业9.5万人。市区11个教育重点项目进展顺利，学前教育三年行动计划全面完成，农村薄弱学校办学条件不断改善，新增国家义务教育均衡县2个。许昌市立医院等7个卫生重点项目、86个基层医疗卫生机构改扩建项目有序推进。实施城乡统一的计划生育奖励扶助制度，连续7次荣获“全省人口计生工作先进市”称号。企业退休人员养老金、城乡居民养老金、新农合和城镇居民医保等标准进一步提高。完成31所农村敬老院“温暖工程”。相继建成两期城市公共自行车系统，更多市民能够就近骑上“小绿”。市区60岁以上老人都可以免费乘坐公交车，成为全省首批“公交优先示范城市”创建城市。加大安居工程建设力度，10328套保障性住房基本建成。南水北调中线工程及配套工程如期建成，丹江水顺利抵达许昌。扎实推进“平安许昌”建设，严厉打击违法犯罪，深入开展安全生产专项治理，社会大局保持和谐稳定。

（七）立行立改转作风，政府自身建设扎实推进。我们以开展党的群众路线教育实践活动为契机，切实加强政府自身建设，集中解决了一批群众反映强烈的“四风”突出问题，制定修订了一批工作制度机制，有力提升了行政效能和发展动能。坚持依法行政，全面推进依法治市，自觉接受人大、政协监督和社会监督，积极认真办理人大代表议案建议和政协提案。扩大政务公开，电子政务、政府网站、市长信箱等民情通道更加畅通有效。强化行政监察和审计监督，制度执行力和约束力得到增强，反腐倡廉工作深入推进，法治政府、高效政府、廉洁政府、服务政府建设取得新的进展。

与此同时，军地军民更加团结，外事、侨务、对台工作全面加强，民族宗教、地方史志、人民防空、体育、档案、地震、气象等工作都取得了新的成绩。

这些成绩的取得，是省委、省政府和市委正确领导的结果，是人大、政协和社会各界关心支持的结果，是全市人民团结拼搏的结果。在此，我代表市人民政府，向工作在全市各条战线上的广大干部群众、解放军指战员、武警官兵，向所有关心、支持许昌发展的各界人士表示衷心地感谢！

我们也清醒认识到，由于宏观经济增速回落，全市经济社会发展与年初预期目标仍有差距，还面临一些突出困难和问题：经济下行压力依然较大，一些主要经济指标增速下滑，生产总值增速低于预期;部分行业和企业运营困难较多，融资困难与金融风险交织并存；经济结构不够合理，服务业增加值占生产总值的比重全省位次靠后，城镇化水平不高，高新技术产业规模相对较小；民生改善任务依然艰巨，公共服务水平与人民期盼差距较大，生态改善和环境保护更加紧迫。同时，政府自身建设仍需加强，政府职能和干部作风尚需加快转变。对此，我们将高度重视，主动作为，采取有效措施认真加以解决。

二、2015年政府工作总体要求和预期目标

今年是全面完成“十二五”规划的收官之年。面对经济发展新常态，我们既要看到社会投资意愿降低、招商引资难度加大，更要看到我市人力资源相对丰富、土地供应空间尚存，对东部产业转移的吸引力仍然很强；既要看到工业增速下滑、企业经营困难较多，更要看到我市产业基础相对较好、有一支优秀的企业家队伍，在新一轮竞争中有一定的比较优势；既要看到服务业比重偏低、消费拉动力量不足，更要看到我市消费结构不断升级、消费需求快速增长，发展空间和潜力巨大；既要看到推动转型升级、转换发展动力的紧迫性和艰巨性，更要看到我市近年来谋划实施了一大批打基础、增后劲的大项目，新的增长点正在逐步形成。总之，我们既要正视困难、保持清醒，更要把握机遇、坚定信心，适应新常态，谋求新发展，努力谱写更加辉煌的新篇章。

今年政府工作的总体要求是：围绕“四个全面”战略布局，贯彻落实省委和市委经济工作会议精神，按照致力打通“六路”、建设“五型许昌”总体思路，坚持稳中求进工作总基调，突出经济发展质量和效益，主动适应经济发展新常态，把稳增长作为首要任务，把转方式调结构放到更加重要位置，全面深化改革，突出创新驱动，争创新的优势，着力改善民生，维护社会稳定，促进全市经济社会持续健康发展。

今年经济社会发展的主要预期目标是：生产总值增长8%以上，地方一般公共预算收入增长10%，工业增加值增长9.5%，固定资产投资增长16%，社会消费品零售总额增长13%，进出口总额增长8%，实际利用外商直接投资增长7%，城乡居民收入与经济同步增长，常住人口城镇化率提高1.8个百分点，居民消费价格涨幅控制在3%左右，节能减排完成省定目标。

实现预期目标，做好今年政府工作，需要切实把握以下5点：一是更加注重职能转变。深入推进简政放权，把政府工作重点转到创造发展环境、提供公共服务、维护公平正义上来。二是更加注重市场运作。遵循市场规律，转变领导方式，充分发挥市场在资源配置中的决定性作用，更多地运用市场的手段破解难题、促进发展。三是更加注重务实推进。加强系统研究，抓住关键环节，选准载体、用好平台、抓牢抓手，努力使推动工作的方法更优、措施更强、成效更好。四是更加注重改革创新。坚决主动、扎实有效推进改革，加快科技进步、扩大开放和全面创新，着力培育形成发展的新活力、新动力、新优势。五是更加注重改善民生。始终坚持发展为民、发展惠民，集中更多财力，强化社会保障，办好民生实事，在改革发展中不断织密民生“安全网”。

三、2015年重点工作

按照市委经济工作会议部署，围绕总体目标，今年突出抓好8项重点工作。

（一）全力保持经济稳定增长。把稳增长摆到更加突出的位置，着力扩投资、促消费，加强经济运行调节，促进经济持续健康运行。

积极扩大有效投资。推进实施“16718”投资促进计划，突出抓好160个市级重点项目，确保完成年度投资700亿元，带动固定资产投资1800亿元。统筹项目建设，以项目扩投资、调结构、促转型、增优势。特别是对三大水利、三国文化产业园等有利长远、事关全局的重大项目，全力保资金、保进度、保质量，圆满完成年度目标任务。全面加强对公共投入项目监管，严格规范公共投入项目招投标，实行公共投入项目财政评审和审计全覆盖，确保财政资金安全高效透明使用；常态谋划优质项目，在国家重点支持的节能环保、生态建设等领域谋划更多项目，争取更多资金支持，并抓好项目前期，力争更多项目早开工、早建成。

加强经济运行调节。完善经济运行监测体系，研究把握宏观政策导向，定期会商研判经济形势，及时有效应对经济波动。坚持服务企业长效机制，定期召开政银企洽谈会、政策咨询会，加强协调沟通对接，及时发现企业运行中苗头性、倾向性问题，积极主动解决企业困难，全面服务经济发展。提高要素保障能力，加强煤电油气运等生产要素调度，重点解决企业资金、土地、用工等瓶颈约束，确保企业特别是百户重点企业稳定运营。

促进消费扩大升级。认真落实国家、省促进消费各项政策措施，多渠道增加居民收入，逐步提高居民收入在国民收入分配中的比重，完善各项社会保障措施，不断提高居民消费能力；积极推进养老健康家政、信息、旅游、住房、绿色、教育文化体育六大消费工程，支持城乡商业服务网点建设，建立推广电子商务公共服务平台，促进信息消费、服务消费和大众消费，满足人民群众个性化、多样化消费，培育一批新的消费热点。

（二）加快优化经济结构。以提高经济发展质量和效益为中心，加快产业结构战略性调整，促进先进制造业、高成长服务业协调发展，加快工业化与信息化深度融合，推动产业结构向高端发展。

加快发展服务业。坚持现代服务业和传统服务业并举、生产性服务业和生活性服务业并重，落实各项优惠政策，实施服务业发展提升计划，放宽市场准入，促进服务业多元发展、创新发展，努力把服务业发展的巨大潜力转化为现实生产力。推动现代物流、信息服务、金融、文化、体育、旅游等高成长服务业提质扩容增效，加快建设中原国际农产品物流基地等项目，构建市区农产品流通三级网络体系，全面提升全国二级物流园区布局城市水平；加快发展科教、商务服务、健康养老等新兴产业，依托生态和产业优势，加快建设鄢陵全国生态休闲养生基地，促进禹州中医药保健产业发展；改造提升传统服务业，创新商业业态、商业模式和服务产品，推动房地产业健康发展，加快商贸流通等传统支柱服务业转型升级；依托商务中心区和特色商业区，坚持城镇化与服务业融合发展，突出抓好楼宇经济和城市综合体建设，实施特色商业街示范创建工程，争取新开工项目30个，完成投资50亿元。研究制定与郑州航空港经济综合实验区融合发展的政策措施，借助航空经济的引领作用，着力发展高端制造业和现代服务业，积极发展航空物流、服务外包等新兴业态，重点培育与航空经济相配套的特色产业链。高度重视电子商务和跨境电子贸易发展，扶持发展一批电子商务产业园和电商企业、“电商村”。

推动工业转型升级。突出主导产业，培育产业集群，着力构建企业集聚、产业集群、发展集约的新格局。推进实施十大产业链发展计划，优先发展电力装备首席产业链，培育壮大新一代信息技术、工业机器人、新能源汽车三大新兴产业链，优化提升生物医药、汽车及零部件、电梯、食品及冷链、超硬材料及制品、再生金属及制品六大优势产业链，推动工业结构优化升级。加快实施120个亿元以上工业结构调整项目，突出抓好100个亿元以上省级工业结构调整项目、25个战略性新兴产业重大研发与产业化项目，争取早日投产达效。引导纺织、建材、有色、煤焦化等传统产业，延链补链，兼并重组，加快改造升级。提升产业集聚区建设水平，加快培育千百亿级产业集群，力争产业集聚区完成固定资产投资1200亿元，规模以上工业企业主营业务收入超过3000亿元。完善产业集聚区生产性、生活性基础设施，实现产业集聚区发展与城市建设有机对接、互促共进。

推动信息化发展。以国家实施“互联网+”行动计划为契机，积极创建“宽带中国”示范城市，促进云计算、大数据、物联网等与现代制造业相结合，加大信息技术在现代物流、工业设计等服务业领域的广泛应用，提高信息化基础支撑能力。实施信息化建设三年行动计划，加快建设智慧城市，重点打造许昌信息产业园和产业集聚区两化融合信息服务平台，实施居民“一卡通”、教育信息化等项目建设，全面提升信息化应用水平。加快发展信息产业，深化与信息化龙头企业合作，着力引进一批高水平的信息化企业和项目，大力发展众品冷链物流等电子商务平台，培育物联网等信息产业新业态。

加快培育重点企业。大力扶持百户重点企业，加强与央企、国内外500强企业合作，推动企业兼并重组，抢抓资本市场改革发展新机遇，加快企业上市步伐，积极推进新三板挂牌工作。引导企业建立完善现代企业制度，树立品牌意识，提高决策水平，加强内部管理，加快转型升级，增强市场竞争能力。全年争创河南省名牌产品10个，河南省质量诚信A等企业6家。

（三）切实提升城镇化发展质量。促进布局、形态不断优化，推动内涵、质量不断提升，使城镇化发展水平与工业化、现代化相适应，让许昌在中原城市群核心城市中更加有位有为。

加强规划编制管理。着力提高规划水平，切实发挥规划的引领、调控和约束作用。科学务实有序编制规划，做到城市总体规划与专项规划、各专项规划之间深度融合、科学匹配，实现公共资源合理均衡配置。加强县（市）城区总体规划、县域村镇体系规划和新农村建设规划编制管理，完善城乡规划体系。

加快城乡一体化示范区建设。按照一二三产业复合、经济生态人居功能复合的理念，完善规划，积极探索，扎实稳妥推进城乡一体化示范区建设。认真实施重点项目推进计划，强力推进中原电气谷、商务中心区、中国中医药产业园建设，争创国家级高新技术产业开发区。理顺示范区行政管理体制，力求统筹、精干、灵活、高效。

增强中心市区承载能力。实施总投资667亿元的178个城建重点项目，完成年度投资190亿元。加快重点区域开发建设，突出抓好护城河水系环通及周边综合开发、中轴线商务中心区景观打造及两侧综合开发等十大城建工程，加快交通、供排水、供热、供电、供气、污水、垃圾处理、夜景亮化、地下空间开发及人防工程等基础设施建设，进一步完善城市功能、提升城市形象。结合35个棚户区改造项目，加强城市综合体建设，完成年度投资42.5亿元。加快国道107许昌境改线、许昌至新郑机场快速通道、中心汽车站改造等项目建设，积极创建国家公交都市。加强城市管理，巩固提升全国文明城市创建成果。

统筹推进城乡发展。加快禹亳铁路灵井至许昌东、许昌东至鄢陵段改建工程，继续推进城市间、城镇间快速通道建设，加强组团内部联系。坚持内涵式发展，统筹推进县（市）城区、中心镇区和新农村建设，完善城乡基础设施和公共服务设施，支持禹州市加快国家新型城镇化综合试点市建设，提升组团城市综合承载能力。加快30个中心镇发展，积极扎实推进新农村建设，加强农村公共服务和社会管理，实行农村垃圾、污水收集处理，完善配套基础设施，创建“美丽乡村”，改善农村居住环境。进一步完善征收拆迁政策，坚持依法、阳光、和谐拆迁，切实维护群众合法权益。围绕“产业为基、就业为本，住房和教育牵动，完善社会保障、农民权益保障、基本公共服务保障”，同步实施户籍、教育、医疗、土地、社保和公共服务等配套改革，有序推进农村人口向城镇流动和农业转移人口市民化。

（四）扎实做好“三农”工作。加快发展农业现代化，转变农业发展方式，积极实施“三大工程”，让农业更强、农民更富、农村更美。

切实稳定粮食生产。围绕国家粮食生产核心区建设，扎实推进高标准粮田“百千万”工程，新建高标准粮田40万亩，建立粮食高产创建示范方37个，新增高效节水灌溉面积16.3万亩、有效灌溉面积10万亩、旱涝保收面积6万亩，粮食总产稳定在280万吨以上。创新土地、道路、电网、水网、林网等公共设施管护机制，保障高标准粮田持久发挥效益。加快推进气象现代化试点市建设，提升气象灾害监测防御能力和服务保障水平。

壮大农业产业集群。加快农业产业化发展，推动农业产业结构调整。深入推进6个省级农业产业化集群建设，加快蜂产品、豆制品、中药材等农业产业化集群培育，认定一批市级农业产业化集群，争取2个以上集群获得省级认定。积极推广种养结合和农牧循环发展模式，持续提升畜牧业现代化水平，力争畜牧业产值占农业总产值的比重达到49%以上。充分发挥农业龙头企业的示范作用，带动小型企业和农户提升生产经营管理水平，实现互惠互利、共同发展。

发展都市生态农业。打造半小时“都市生态农业圈”、特色都市生态农业园区和示范性都市生态农业基地，积极申报国家休闲农业与乡村旅游示范点，加快发展旅游观光农业。开展园艺作物标准园创建活动，加强农产品质量安全可追溯体系建设，提高蔬菜标准化生产水平。

扩大新型经营主体。大力培育专业大户、家庭农场、农民专业合作社和农业产业化龙头企业等新型农业经营主体，提升农业集约化、专业化、组织化经营水平。新培育农民合作社400家、家庭农场200家，新增一批市级农业产业化龙头企业。

持续改善基础条件。认真落实良种补贴、农机具购置补贴、粮食最低收购价、生猪良种补贴等惠农政策。加快水利基础设施建设，完成8条中小河流治理、清潩河马岗闸等除险加固、第五批“小农水”重点县建设任务，治理水土流失面积30平方公里。继续实施农村公路“三年行动计划”，提升改造农村公路520公里、桥梁3850延米。加强农村水电气等设施建设，实施农配网改造升级工程，完成7.4万户农村“低电压”治理，新解决18.7万农村人口的饮水安全问题，搞好农村户用沼气和大中型沼气工程建设，鼓励发展农村集中供气。做好扶贫开发工作，实施20个村整村推进，通过产业开发实施精准扶贫，新解决2.5万人脱贫问题。

（五）深入推进改革创新。坚持问题导向，聚焦影响经济社会发展的突出障碍、聚焦群众反映强烈的突出问题，推动重点领域和关键环节改革取得新突破。

全面深化各项改革。深化行政审批制度改革。继续压缩、取消、下放市级行政审批事项，优化审批流程，缩短审批时限，向社会公示，请群众监督；积极推进制定政府权力清单和责任清单，切实做到法无授权不可为、法定职责必须为。加快财税体制改革。认真落实新《预算法》，改进预算管理制度，完善政府预算体系，大力推行预算公开，使预算管理全面规范、公开透明；加强政府债务管理，规范政府融资平台运作，把政府性债务分类纳入全口径预算管理；盘活财政存量资金，提高资金使用效率；做好转移支付改革政策的对接落实，完成“营改增”扩面、资源税从价计征、消费税调整等税制改革任务。积极推进投融资体制改革。采取投资补助、资本金注入等办法，引导社会资本设立股权投资基金、投向重点项目；支持在基础设施、现代产业等重点领域发起设立创业投资基金和产业投资基金，特别是抓住我省设立新型城镇化发展母基金的机遇，创造条件，加强探索，积极设立子基金；积极推广政府和社会资本合作模式，加快基础设施、公用事业等领域建设。深化医药卫生体制改革。积极推进县级公立医院综合改革，拓宽社会办医领域，支持社会资本举办非营利性医疗机构；完善城乡医保体系，健全重特大疾病保障制度，推进基本公共卫生服务均等化。加快农村改革。继续做好农村土地承包经营权确权登记颁证试点工作，开展农村土地承包经营权抵押贷款试点，构建农村集体产权交易平台，基本实现县级全覆盖。同时，全面完成市、县政府机构改革，有序推进党政机关公务用车、企事业单位改制、教育、环保、文化、城管等方面的改革。

完善要素保障机制。健全完善人力资源、土地交易市场，探索培育资金、资本等其它各类要素市场。围绕服务实体经济，推进金融改革，鼓励民间资本进入金融机构改革重组，推动具备条件的民间资本发起设立中小型银行等金融机构，引导村镇银行扩大业务覆盖面，积极推进农商行组建，规范发展融资租赁、典当行等准金融机构。优化信贷结构，提高资金使用效率，全年新增贷款160亿元、实际融资300亿元。坚持并完善企业发展互助资金等制度机制，防范化解企业财务风险，切实缓解企业融资难、融资贵问题。建立健全城镇低效用地再开发激励约束机制，全力推进城乡建设用地增减挂钩和人地挂钩试点工作。

全面推进科技创新。深入开展国家创新型试点城市、知识产权示范城市创建工作，鼓励大众创业、万众创新，增强自主创新能力，促进经济优质、高效发展。深化科技体制改革，突出企业创新主体地位，建立主要由市场决定科技创新项目和资金分配、成果评价机制，加强知识产权运用与保护，营造鼓励创新、宽容失败的氛围。突出抓好产业创新，围绕重点产业，选择一批共性关键技术重点攻关，提升产业核心竞争力。加快科技企业孵化器及企业研发平台等载体建设，引导企业加大创新投入，依托重点骨干企业，选准项目，积极推介，力争年内实施省级重大科技专项和高新技术产业化项目3—5个，新增高新技术企业8家以上。继续做好发明专利的申报工作，加快专利转化平台建设，促进专利技术实现产业化。

（六）不断扩大对外开放。围绕优化结构、促进转型，积极实施开放招商、承接产业转移，加快构建活力强、效率高的开放型经济新体系。

提高招商引资水平。充分利用国际、国内两个市场，拓宽开放空间，推动全方位、多领域开放招商。大力实施“6113”招商引资行动计划，谋划推进600个重点招商项目，推动100个亿元以上签约项目落地，全年合同引进市外资金1300亿元。突出抓好驻地招商和委托代理招商，以产业集聚区为平台，围绕主导产业延链补链，开展专题招商，各驻地招商点年内至少引进2个5亿元以上主导项目。突出集群式引进、专题性推介、区域性对接，用好三国文化旅游周、花木交易博览会、钧瓷文化节等各类招商平台，着力在高成长服务业和十大产业链招商引资上实现新突破。

大力实施“走出去”战略。积极主动融入国家“一带一路”战略，打造开放的高地，增创新优势。积极推动企业参与国际合作与竞争，鼓励农业“走出去”，扩大对外承包工程和劳务合作，支持涉外企业积极开拓海外市场，扩大我市特色优势产品出口规模。加大对“走出去”企业金融支持，鼓励使用人民币对外贷款和投资，加强境外企业风险防范和安全生产。

加快开放载体平台建设。进一步提升“大通关”建设水平，尽快实现许昌出入境检验检疫局高效运转，积极推进许昌海关筹建工作。继续抓好我市国家级、省级出口基地建设，促进发制品等相关企业转型升级。提高城乡一体化示范区服务外包示范园区建设水平，做好长葛市蜂产品出口基地创建及管理工作。

优化开放投资环境。清理规范有关优惠政策，在营造良好市场环境、提高办事效率和服务水平上下功夫。围绕引资信息处理和受理服务、联审联批和代理服务、投诉权益保护，探索建立招商全链条服务框架，促进投资贸易便利化。着力构建社会诚信体系，加快政务信用记录建设，建立失信行为联合惩戒机制。

（七）切实加强生态环境建设。以“美丽许昌”建设为统领，围绕天蓝水净地绿的目标，着力解决群众最关心的环境问题，努力让美丽与发展同行，让生活与自然同在。

大力实施“三大工程”。实施“蓝天工程”，积极发展清洁能源，推进大气污染联防联控，实施机动车排气污染监测和环保标志管理制度，完善重污染天气监测预警体系和应急预案。实施“碧水工程”，围绕水生态文明试点市建设和水系连通工程，以清潩河流域水环境综合整治为重点，加强水污染防治，持续推进污水处理厂建设，深化工业污染防治，落实生态补偿机制，进一步改善水环境质量。实施“乡村清洁工程”，开展农村环境综合整治，加强农村面源污染治理，加快规模化畜禽养殖污染治理项目建设，力争完成7个省级生态村和1个省级生态乡（镇）创建。支持鄢陵县进一步创建国家级生态县。

加强林业生态建设。以“四海”工程为重点，搞好三大水利项目配套绿化、美化，打造“河畅、湖清、水净、岸绿、景美”的林水一体生态景观系统，推进城郊、水系、生态廊道等林业生态建设，着力推进林业生态建设与城镇化建设相结合，切实提高宜居水平。加强森林资源保护，完成造林任务11万亩。支持禹州市申报省级以上废弃矿山治理项目，加快西部山区生态修复。

强化节能减排。加强污染物排放总量控制，继续实施建设项目环境准入禁止、限制区域和项目名录，控制“两高一资”、产能过剩、布局不合理、低水平重复建设项目。提高资源节约集约利用、企业清洁生产水平，深入实施工业、建筑、交通运输等七大节能降耗行动计划，全年万元生产总值能耗下降率达到省定目标。推进建筑垃圾、生活垃圾资源化利用。落实最严格水资源管理制度，深入推进国家节水型城市建设。加强环境监管，全面落实主要污染物排放总量预算管理、排污权有偿使用及交易管理等制度。

（八）着力保障和改善民生。继续实施一批民生工程，促进各项社会事业繁荣发展，在学有所教、劳有所得、病有所医、老有所养、住有所居上取得新进展，不断增进民生福祉。

突出抓好创业就业。以创建国家级创业型城市为载体，深入实施全民技能振兴工程和职教攻坚二期工程，加强公共就业服务和人力资源开发，完成各类技能培训15万人次以上。鼓励支持初次创业和小微企业发展，激发创业创新活力。做好高校毕业生、农村转移劳动力、退役军人、就业困难人员等重点群体就业工作，实现新增城镇就业6.6万人、高校应届毕业生综合就业率保持在90%以上，动态消除城镇“零就业”家庭。落实好最低工资制度，集中治理欠薪、打击非法中介。让群众的创业就业之路更加简便、更加顺畅。

提高社会保障能力。坚决兜住兜牢民生底线，完善社会保障体系，加强城乡低保规范管理，提高企业退休人员养老金、城乡低保、五保供养标准，城乡居民基本养老保险基础养老金由60元提高到80元，按时足额发放各项保障资金。加大社会救助力度，做好救灾救济工作，关爱农村留守人员，切实做到求助有门、受助及时。加强保障性安居工程建设，改善困难家庭的住房条件。促进残疾人、妇女儿童、红十字、老龄等事业发展，加快建设县级社会福利中心。让人民群众生活得更加幸福、更有尊严。

加快发展社会事业。努力办好人民满意的教育，让每个人都能通过教育改变自身命运。实施第二期学前教育三年行动计划，以中小学标准化建设为抓手，以“全面改薄”和扩充城镇义务教育资源为载体，加快推进义务教育均衡发展，支持普通高中多样化发展，加快发展现代职教体系。合理配置城乡教育资源，保障进城务工人员子女入学，让所有孩子都能接受公平有质量的教育；加快建设寄宿制学校，积极引进名校资源，各县（市、区）年内新增2所以上寄宿制学校，安排不少于2个社会办学项目，满足多样化、多层次教育需求。提升群众健康水平，健全医疗卫生服务体系，提高基层医疗机构诊疗水平；开展城乡居民大病保险，城乡居民医疗保险财政补助标准从每人每年320元提高到380元，基本公共卫生服务经费财政补助标准从人均35元提高到40元，免费为城乡居民提供12类45项基本公共卫生服务；全面实施县级公立医院综合改革，破除以药补医，努力减轻群众就医负担；加快发展中医药事业，加强重大疾病防控和重特大疾病救助。依法处置医疗纠纷，构建和谐医患关系。继续做好计划生育工作，促进人口长期均衡发展。加快推进市级全民健身中心项目建设，广泛开展全民健身运动，提高人民群众身体素质。深入实施文化振兴计划，组织实施广播电视户户通、舞台艺术送农民等文化惠民工程，着力抓好11个文化旅游产业重点项目，加快建设市科普教育基地等公共文化服务设施，支持民办博物馆、电影院发展。让城乡居民享有的公共服务更加全面、更加优质。

加强和创新社会治理。深化社会组织管理制度改革，加快行业协会商会与行政机构脱钩。支持群团组织依法参与社会治理，发展专业社会工作、志愿服务和慈善事业。深化平安许昌建设，树立“大平安”理念，加强对经济运行、企业债务以及社会稳定等方面的风险防控。高度重视、积极稳妥地化解和处置非法集资，确保不发生区域性系统性金融风险和群体性极端事件。落实“发放明白卡、签订承诺书、建立黑名单”制度，统筹做好煤矿、消防、交通、食品药品等重点领域和关键环节的安全监管，推行执法人员网格化管理全覆盖，坚决遏制重特大事故发生，尽最大能力保护人民群众生命财产安全。落实社会稳定风险评估和包案、接访制度，深入排查化解矛盾纠纷，全面推行依法逐级走访，组织开展积案攻坚，妥善解决信访突出问题。加强社会治安综合治理，完善立体化防控体系，推进“一村一警”长效机制建设，抓好重点区域治安防控，严厉打击各种违法犯罪活动。让广大群众的生产生活更加安全、更加祥和。

着力办好民生实事。今年，我们在实施各项民生工程的基础上，重点办好10件民生实事：一是完善农贸市场体系；二是改善群众就医条件；三是加大中小学校建设力度；四是抓好安居工程建设；五是实施护城河环通工程；六是建成三国文化演艺中心；七是改善公共交通服务；八是更新环卫设施设备；九是推进数字校园建设；十是加强小区物业管理。这10件实事，事关民生福祉，群众翘首以盼，无论有再大困难，年底都要坚决完成。让人民群众真正感到更加实惠、更加满意。

继续加强国防和民兵预备役建设，促进军民融合发展。支持工会、共青团、妇联、工商联等人民团体发挥桥梁纽带作用。切实做好外事、侨务、港澳、对台工作。继续做好民族、宗教、地方史志、新闻出版、地震、档案、社科研究、对口援疆等工作。

｜今年，我们将在完成“十二五”经济社会发展目标任务的同时，用改革的精神、创新的理念认真编制“十三五”发展规划，谋划好未来五年的发展蓝图，尤其要通过深入调研、科学论证，谋划一批重大项目，争取能够纳入国家、省规划盘子。

四、切实加强政府自身建设

扎实推进法治政府、创新政府、高效政府、廉洁政府、服务政府建设，切实增强政府执行力和公信力，全面提升政府治理水平。

（一）坚持依法行政，着力建设法治政府。深入推进依法治市，弘扬法治精神，树立法治信仰，以法治引领和规范各项工作。要尊法学法守法用法，依法全面履行职责，用法治的思维解决社会热点难点问题。依法接受人大及其常委会法律和工作监督，主动接受政协民主监督。全面落实行政执法责任制，一切违法违规行为都要追究，一切执法不严不公现象都必须纠正。

（二）坚持改革创新，着力建设创新政府。面对新任务，适应新常态，加强学习研究，把握发展规律，努力克服“换挡焦虑”、“本领恐慌”，用改革的办法破除体制机制障碍，用创新的举措开创加快发展的新局面。加强新型智库建设，建立健全决策咨询制度，积极推进决策科学化、民主化，以科学决策引领创新发展。

（三）坚持转变作风，着力建设高效政府。扎实开展“三严三实”专题教育，坚持不懈纠正“四风”突出问题，着力形成改进工作作风的长效机制。始终把抓落实摆到更加突出位置，以抓铁有痕的作风，以钉钉子精神，加压使劲，久久为功，善做善成。切实加强督促检查，提升行政效能，推动工作落实，注重工作实效。坚决纠正不作为、乱作为，坚决克服懒政、怠政，坚决惩处失职、渎职，坚决做到政令畅通、令行禁止。

（四）坚持清廉为政，着力建设廉洁政府。严格落实廉政建设责任制和主体责任，强化“一岗双责”，把廉洁从政贯彻到政府工作各个环节。以权力“瘦身”为廉政“强身”，紧紧扎住制度“围栏”，坚决打掉寻租空间，努力铲除腐败土壤。加强行政监察和审计监督，对公共资金、公共资源、国有资产严加监管。厉行勤俭节约，反对奢侈浪费，把有限的资金更多用于发展经济和改善民生。始终保持反腐高压态势，严厉查处各种腐败行为。

（五）坚持执政为民，着力建设服务政府。进一步规范审批行为，推行一口受理、限时办理、透明办理和网上办理。改进服务方式，加大政府购买第三方基本公共服务力度。进一步提高公务员素质，始终牢记为民宗旨，忠于职守、尽职尽责，决不允许弄权渎职，决不允许与民争利！

各位代表！站在历史新起点，面对人民新期待，应对经济新常态，我们深感责任重大、使命光荣。让我们在省委、省政府和市委的正确领导下，紧紧依靠全市人民，振奋精神，真抓实干，克难攻坚，为建设“五型许昌”、实现“富民兴许”而努力奋斗！
